# Multifaceted Impacts of Plant-Beneficial *Pseudomonas* spp. in Managing Various Plant Diseases and Crop Yield Improvement

**DOI:** 10.1021/acsomega.3c00870

**Published:** 2023-06-16

**Authors:** Najaf Mehmood, Mahnoor Saeed, Sana Zafarullah, Sajjad Hyder, Zarrin Fatima Rizvi, Amjad Shahzad Gondal, Nuzhat Jamil, Rashid Iqbal, Baber Ali, Sezai Ercisli, Muhammed Kupe

**Affiliations:** † Department of Botany, Government College Women University Sialkot, Sialkot 51310, Pakistan; ‡ Department of Plant Pathology, Bahauddin Zakariya University, Multan 60000, Pakistan; § Department of Botany, University of the Punjab, Quaid-i-Azam Campus, Lahore 54590, Pakistan; ∥ Department of Agronomy, Faculty of Agriculture and Environment, The Islamia University of Bahawalpur Pakistan, Bahawalpur 63100, Pakistan; ⊥ Department of Plant Sciences, Quaid-i-Azam University, Islamabad 45320, Pakistan; # Department of Horticulture, Faculty of Agriculture, Ataturk University, Erzurum 25240, Türkiye; ∇ HGF Agro, Ata Teknokent, Erzurum TR-25240, Türkiye

## Abstract

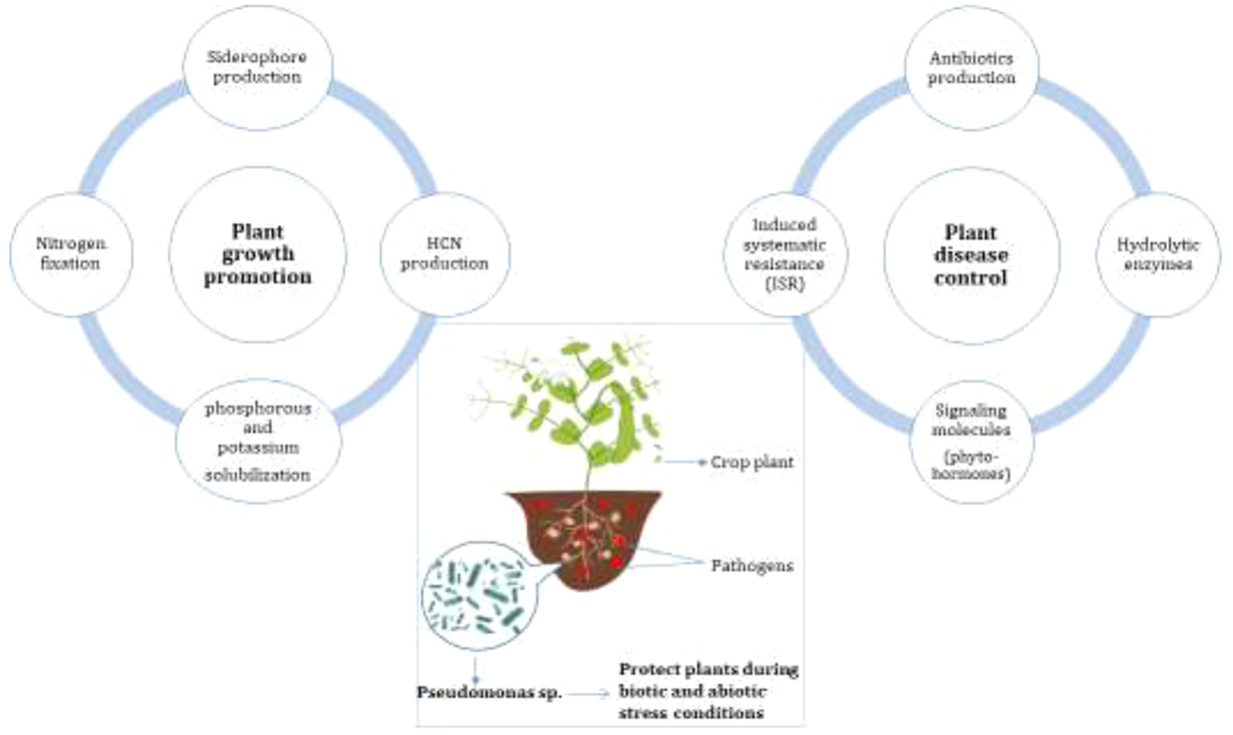

The modern agricultural system has issues with the reduction of agricultural productivity due to a wide range of abiotic and biotic stresses. It is also expected that in the future the entire world population may rapidly increase and will surely demand more food. Farmers now utilize a massive quantity of synthetic fertilizers and pesticides for disease management and to increase food production. These synthetic fertilizers badly affect the environment, the texture of the soil, plant productivity, and human health. However, agricultural safety and sustainability depend on an ecofriendly and inexpensive biological application. In contrast to synthetic fertilizers, soil inoculation with plant-growth-promoting rhizobacteria (PGPR) is one of the excellent alternative options. In this regard, we focused on the best PGPR genera, *Pseudomonas*, which exists in the rhizosphere as well as inside the plant’s body and plays a role in sustainable agriculture. Many *Pseudomonas* spp. control plant pathogens and play an effective role in disease management through direct and indirect mechanisms. *Pseudomonas* spp. fix the amount of atmospheric nitrogen, solubilize phosphorus and potassium, and also produce phytohormones, lytic enzymes, volatile organic compounds, antibiotics, and secondary metabolites during stress conditions. These compounds stimulate plant growth by inducing systemic resistance and by inhibiting the growth of pathogens. Furthermore, pseudomonads also protect plants during different stress conditions like heavy metal pollution, osmosis, temperature, oxidative stress, etc. Now, several *Pseudomonas*-based commercial biological control products have been promoted and marketed, but there are a few limitations that hinder the development of this technology for extensive usage in agricultural systems. The variability among the members of *Pseudomonas* spp. draws attention to the huge research interest in this genus. There is a need to explore the potential of native *Pseudomonas* spp. as biocontrol agents and to use them in biopesticide development to support sustainable agriculture.

## 1 Introduction

Agriculture is very important for animal and human food in the world,^1^ but a large number of crop losses occur each year due to pathogen invasion, which involves a wide variety of pathogens ranging from viruses to prokaryotic bacteria, nematodes, eukaryotic fungi, and oomycetes.^2,3^ On the other hand, it is predicted that the world’s population will reach near 9 billion in 2050, which would generate more burden on food production, space, and the environment.^4^ Since the beginning of the agricultural system, people also have been fighting against the various plant diseases, which significantly has led to the deployment and invention of synthetic fertilizers used to improve crop yield and control disease. These synthetic fertilizers pose a severe threat to the soil biota by disturbing environmental nutrient cycles, disrupting the biological communities existing in the environment, and having adverse health consequences.^5^ The environmental protection and health issues of these harmful chemical materials have increased the necessity of searching for substitutions to control plant diseases and pests.^6^ The application of biocontrol plant-growth-promoting rhizobacteria (PGPR) has been proven to be ecofriendly and cost-effective and has a significant role in managing various diseases, as well as improving plant growth and productivity.^7^

One group of very significant bacteria that has become a focal point of research on biological control of plant diseases is the genus *Pseudomonas* (ubiquitous, rod-shaped γ-proteobacteria and Gram-negative bacteria having polar flagella).^8^ Using multilocus sequencing techniques, it has been estimated that the *Pseudomonas* genera is comprised of more than 100 species, including groups and subgroups.^9^ The genome size of *Pseudomonas* spp. usually varies from 4.6–7.1 Mb and possesses 57.8–66.6% GC content with 4237–6396 expected genes.^10^*Pseudomonas* spp. has valuable applications in the biotechnology, biological control, bioremediation, and plant growth promotion (PGP).^11,12^

The *Pseudomonas* genus is mostly used as a crop inoculant because of its abundant occurrence and versatile metabolic ability, which promote plant growth in many ways, including ACC deaminase activity, nutrient uptake, and antioxidant activities.^13^ The particular strains of *Pseudomonas fluorescens* have been used as seed inoculants on various crop plants to stimulate growth parameters and enhance crop output. These bacterial agents quickly inhabited roots of potato, radish, and sugar beet, which significantly increased the plant yield.^14^*P. fluorescens* may assist in stimulating cabbage growth-related processes, especially by promoting seedlings’ rapid growth and reducing transplant shock.^15^

Among the nutrients, nitrogen (N) plays an important role in the growth and productivity of the plants.^16^ Soil microbes transform atmospheric nitrogen into ammonia using enzyme nitrogenase. The members of *Pseudomonas* spp. have a reported nitrogen fixation ability.^17^ Similarly, phosphorus is the second most important nutrient for plant development and growth, but it is present in an unavailable form. Microbes associated with rice, wheat, maize, and legume crops are mainly reported with a P-solubilization ability, while some others belong to *Burkholderia*, *Enterobacter*, *Halolamina*, *Pantoea*, *Pseudomonas*, *Citrobacter*, and *Azotobacter* are also described.^18^ Along with this, potassium is also required for the development and establishment of plants. Various studies have highlighted the role of *Pseudomonas* spp. in solubilizing the complex soil minerals including potassium aluminum silicate.^19,20^

*P. fluorescens* and *Pseudomonas aeruginosa* are the Gram-negative bacteria that produce hydrogen cyanide (HCN), a secondary metabolite involved in disease inhibition.^21^ The antagonistic potential of *P. fluorescens* is exacerbated by the formation of HCN, which is promoted by iron availability in wet, O_2_-depleted soils. Iron is found in an abundant form but is not available to the plants^22^ and microbes. To get iron for growth and development, some bacteria manufacture low-molecular-weight iron complexes known as siderophores,^27^ which are helpful in limiting the phytopathogens.^23^

In the rhizosphere, bacteria produce phytohormones such as auxin, gibberellins, abscisic acid, ethylene, and cytokinins that stimulate plant growth.^24^ Similarly, antibiotics are small-molecule toxins that may kill or resist the development of other microorganisms.^25^ Several rhizospheric bacteria have also been shown to produce antibiotics as well as toxins^26^ including amphisine, phenazine, 2–4-diacetylphloroglucinol, pioluteorina, pyrrolonitrin, hydrogen cyanide (HCN), oomycine, polymyxin, circulin, colistin, tensin, tropolone, and cyclic lipopeptides.^27^*Pseudomonas* spp. usually produce antibiotics that indirectly support plant growth stimulation.^28^ Various studies have highlighted the active role of *Pseudomonas* spp. in suppressing various fungal pathogens,^29−31^ inducing systemic resistance,^32^ and producing gibberellic acid (GA) and jasmonic acid (JA) signals.^33^

*Pseudomonas* spp. are extensively studied in disease management strategies. The most common compounds that take part in biocontrol mechanism include phenazine-1-carboxamide, lipopeptides, 2, 4-diacetylphloroglucinol (DAPG), pyrrolnitrin, pyoluteorin, and phenazine-1-carboxylic acid (PCA).^11^*Pseudomonas* strains survive well in stress conditions and are very beneficial in the control of several diseases triggered by fungal phytopathogens,^43^ mainly damping-off disease caused by *Pythium spp.*,^34^*Fusarium solani* causing okra root rot,^35^*Rhizoctonia solani* associated with *Rhizoctonia* root-rot,^36^ and foliage blight disease caused by *Phytophthora nicotianae*.^37^

Plants’ ability to defend themselves is strengthened through priming as an adaptive technique. Activation of the induced defense mechanism is one of this phenomenon’s characteristics. Pathogens, arthropods, and abiotic signals operate as warning signs that create a prime. The plant may experience changes at the physiological, transcriptional, metabolic, and epigenetic levels in response to the stimulation. Priming functions as a kind of immunological memory for the plant because it can persist a long time, be maintained throughout the plant’s life cycle, and be passed down to next generations.^38^ The genus *Pseudomonas* holds complex enzymatic system and produces hydrolytic enzymes that play a vital role in the suppression of various plant diseases.^11^ Some important members of *Pseudomonas*, including *P. aeruginosa*, *P. fluorescens*, and *Pseudomonas stutzeri*, secrete chitinases and are considered as good biocontrol agents. *Pseudomonas putida* produces various mycolytic enzymes, including cellulase, lipase, protease, chitinase, and amylase, and develops innate resistance against *Fusarium* wilt disease in *Solanum lycopersicum*.^39^

Pseudomonads also secrete specific biosurfactants, especially biological rhamnolipids (RLs).^40^ RLs have antimicrobial activities against plant pathogens, control a variety of plant disease by stimulating plants defense response,^41^ and also possess efficient remediation abilities.^42^ As RLs breakdown the plasma membrane of fungal zoospores, the use of rhamnolipid-producing *Pseudomonas* spp. could help to manage damping-off disease in chilli and tomato plants.^43^ They also stimulate the immune system and develop local resistance in grapeseed against *Botrytis cinerea* and *Leptosphaeria maculans*.^44^

Pseudomonads aid plants growing under diverse abiotic pressure by producing exopolysaccharides.^45^ For example, *Pseudomonas azotoformans* and *Pseudomonas argentinensis* are reported to reduce the saline stress in the *Brassica juncea*, improve yields, and could be used as a good biofertilizer.^46^*Pseudomonas aeruginosa* has been reported to enhance the growth of *Vigna radiata* (mung beans) plants under drought conditions,^47^ while *Pseudomonas poae* and *P. fluorescens* species increased the number and plant biomass of petunia flower under drought and low-nutrient conditions.^48^

Some *Pseudomonas* spp. have also been promoted as commercial products of plant growth promotion (PGP) and biological control, such as biopesticides and biofertilizers.^49^ The Bio Save 10 LP and Bio Save 11 LP products obtained from *Pseudomonas syringae* strain ESC-10 are employed for the control of postharvest diseases of citrus fruit, apple, potatoes and stone fruits. Similarly, *P. fluorescens* A506 is used to make Blight Ban A506, which is registered as a biopesticide for the suppression of frost damage on pome fruits, peach, cherry, potato, strawberry, almond, and tomato. Blight Ban A506 is also used for the suppression of fire blight.^50^ Thus, multifaceted impacts have created a need to explore the potential of locally occurring *Pseudomonas* spp. against broad range of phytopathogens and bacteria-based bioproduct development.

## 2 The Current Situation of Plant Diseases

Plant pathology faces lots of ever-growing challenges. In the present era, the crop yield has become challenged because of threats from plant diseases and the huge application of synthetic fertilizers. Crop productivity declines due to high plant disease rates that are often between 21 to 30% worldwide in some of the most important crops.^50^ Achieving the basic requirements of a rising population with little resources and without harming the atmosphere is biggest challenge for researchers and farmers.^51^ Ray and co-workers reported that depleting arable lands and less available resources decrease the ability the agricultural yield to flourish.^52^ Moreover, intensification, monocultures, and other high resource inputs (water, pesticides, and chemical fertilizer) in agriculture aim to attain maximum plant yield as the sole target, thus facilitating the progression of many plant diseases worldwide.^53^ Synthetic pesticides do not decompose into simpler and harmless elements, and ultimately they persist in the soil as lethal remains often associated with health problems in humans.^54^ Lucas suggested that several phytopathogens have developed resistance to chemical pesticides that have been in usage for a long period of time.^55^ Therefore, few plant diseases of economic significance have become very problematic to handle due to depleting essential resources;^56^ food market globalization and intensive crop production practices have worsened the condition.^57^

The rapidly increasing demand for substitutions to chemical fertilizers has given chances for the use of biocontrol practices.^58^ Providing adequate, nontoxic, and healthy food for society is always the most essential task of plant disease management. Therefore, plant disease management must ensure a good food supply by increasing the agricultural yield, decreasing food pollution, and also providing a variety of foods at reasonable prices.^59^ Furthermore, raising the public awareness about environment and human health concerns associated with man-made toxic chemicals is also causing a shift toward the highly sustainable management applications that rely less on artificial fertilizers and have no bad effects on the environment and natural resources.^60^ However, the utilization of agrarian products as phyto-stimulators, biocontrol agents, and biofertilizers with some suitable crop management practices is a fantastic choice in sustainable agricultural applications due to its environmentally safe nature. In this context, an effective and ecofriendly strategy is to use *Pseudomonas* spp. as one of the effective choices for the improvement of the plant growth and disease suppression to support sustainable agriculture.

## 3 *Pseudomonas* spp.: An Extremely Diverse Group

*Pseudomonas* spp. are anaerobic, nonendosporic, motile, and rod shaped; they has one or many flagella, are Gram-negative in nature, and belong to the proteobacteria group.^61^ They are a saprophytic in nature, found in water, soil, and moist areas.^62^ They have been identified to act as both plant growth promoters (*Pseudomonas fluorescens*) and plant pathogens (*P. syringae*).^63^ The number of *Pseudomonas* isolates may change with the passage of time; the newest molecular diversity analysis (MDA) represents 144 species and remains as the largest group of microbes.^64^ However, the widespread occurrence of *Pseudomonas* spp. shows their adaptability through the physiological, environmental, and molecular diversity.^65^ The phylogenetic relatedness of *Pseudomonas* strains within the genus can be determined through the sequence analysis of conserved genes.^66^ According to Höfte and Altier, the biocontrol abilities of bacteria are strain-dependent and cannot link with phylogenetic variations.^67^ They directly act on the physiology, growth, and nutritional status of the plant they inhabit. *Pseudomonas* spp. also has the potential to withstand the high temperature up to 35–38 °C.^68^

**Figure 1:**
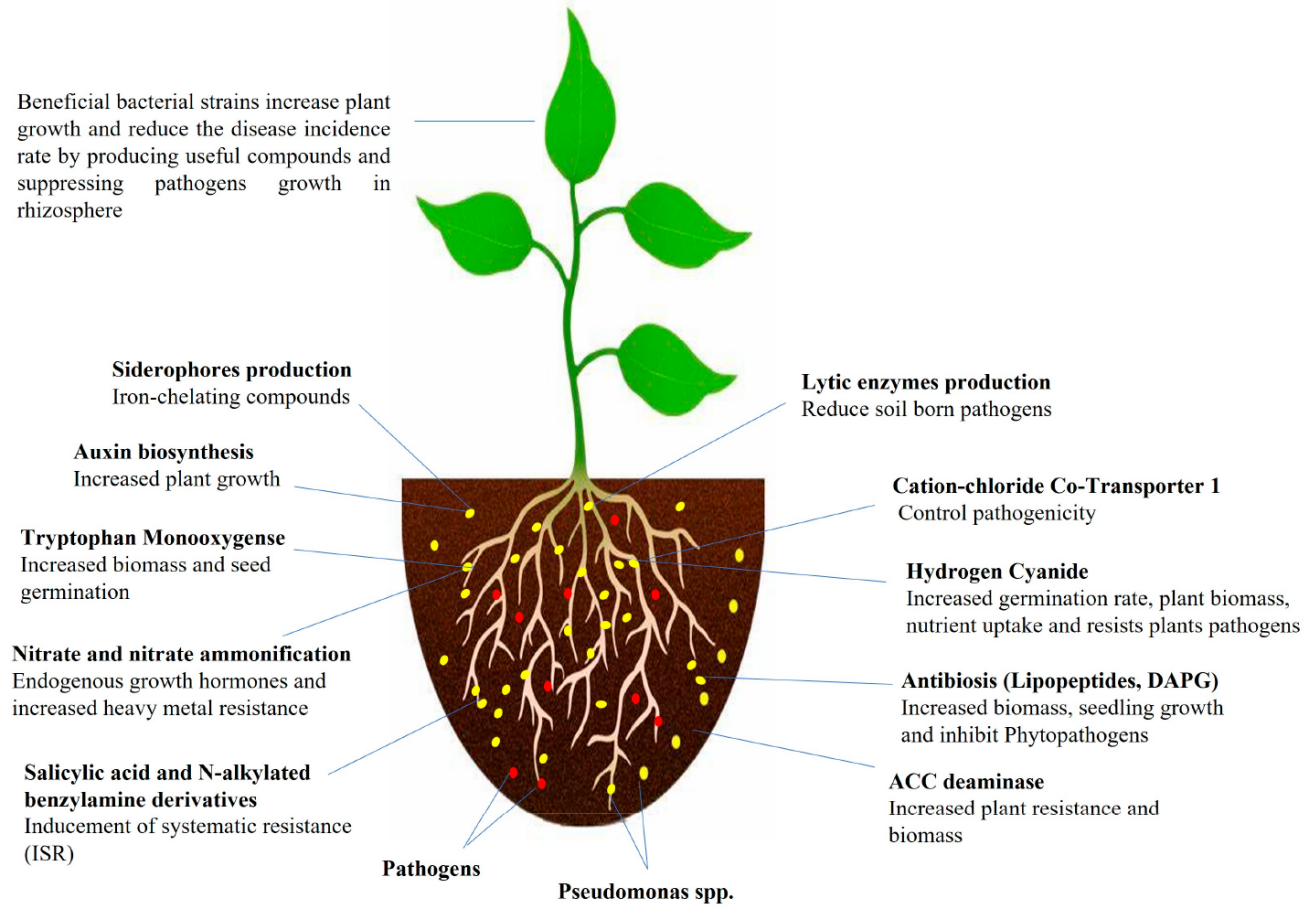
Schematic representation of the mechanisms of action of *Pseudomonas* spp. in the rhizosphere.

Numerous *Pseudomonas* spp. including *P. aeruginosa*, *Pseudomonas chlororaphis*, *P. putida,* and *P. fluorescens* are well-known for their capacity to improve plant growth and lessen a variety of plant diseases.^69^ These are extensively found to be associated with the roots of plants and give advantage to plants by fending off plant pathogens. Several traits of *Pseudomonas* spp. enable them to serve as plant growth promoters and biocontrol agents. These traits include aggressive rhizosphere competence, quick colonization, formation of several root exudates, bioactive substances (such as vitamins, VOCs, siderophores, and antibiotics), and stress responses^70^ (Figure 1). In case of *P. fluorescens*, genes of the tryptophan synthase-α chain (trpA) and tryptophan synthase-β chain (trpB) generate indole as an intermediate product, which via the expression of tryptophan 2-monooxygenase (iaaM) catalyzes indole-3-acetamide to IAA using iaaH,^71^ increasing plant growth. Some plant hormones, such as indole acetic acid (IAA), cytokinins, gibberellins, and ethylene production inhibitors are produced by fluorescent *Pseudomonas*, which help to improve the plant roots capability to absorb water and certain nutrients.^72^

## 4 *Pseudomonas* spp. as Plant-Growth-Promoting Rhizobacteria

Plant-growth-promoting rhizobacteria (PGPR) are beneficial rhizospheric microbes that are free-living and engaged in promoting plant growth and development. PGPR inhabit the rhizosphere and roots of the plant, growing in the spaces between the root hairs and rhizodermal tissues.^73^ PGPR are not only linked with the root to have advantageous impacts on plant growth and development but are also responsible for the obliteration of plant pathogenic microorganisms. These bacteria are being used at the field level and have long been well-known to increase agricultural yield in normal and stressed soils due to their ecofriendly nature.^74^ They enhance the plant growth through establishing induced systemic resistance (ISR), competitive omission, and antibiosis and defending the plants against biotic agents.^75^ They raise the level of antioxidant enzyme and protecting the plant cells from an oxidative stress.^76^ Thus, PGPR act as one of the most significant ingredients in the formulation of biofertilizers.

The rhizosphere is a soil zone under the influence of plant roots that is a challenging and heterogeneous environment shaped by plant rhizo-deposition^77^ and colonized by many microorganisms, including plant-associated *Pseudomonas* spp. *Pseudomonas* spp. are very ubiquitous microbes in the rhizospheric zone that possess the characteristics to be well-suited to thePGPR category (Figure. 1).^78^*Pseudomonas* spp. have various PGPR qualities, including (i) fast in vitro growth and the capacity for biomass production; (ii) colonizing and dominating in the spermosphere, rhizosphere, and inside the plant’s body; (iii) the production of bioactive metabolites (antibiotics, growth-promoting substances, siderophores, and volatiles); (iv) quick use of seed and root exudates; (v) violent competition with other microorganisms; and (vi) an environmental stress resistance ability. Due to the production of the above stated compounds, they are responsible for the plant growth promotion and innate resistance of some soils to toxic pathogens.^79^

It is well proven that the plant growth is enhanced through the application of these beneficial bacterial populations (Table 1). The *P. fluorescence* (FLPs) strain was used as an inoculant in the combination of microbial rich fertilizer to improve the growth parameters and yield of chickpea plants.^80^ Similarly, *P. aeruginosa* isolated from rhizospheric soil, shoots, and roots of sugar cane provides a remarkable improvement in fresh and dry mass of sugar cane.^62^ Specific strains of *P. fluorescens* and *P. putida* have been used as seed inoculants on crop plants to stimulate their growth and enhance crop yields. *P. aeruginosa* was utilized in along with *B. subtilis* and *T. harzianum* to combat *Sclerotinia sclerotiorum*, leading to plant health improvement by eliciting systemic resistance and proteome level changes.^81^*P. azotoformans* considerably enhanced plant nutrient (calcium, manganese and potassium) uptake in contrast to the mutant strain of *P. azotoformans*.^82^

**Table 1:** Role of *Pseudomonas* as a Better Plant Growth Promoter

bacterial strain	targeted plant species	beneficial impact on plant	references
*P. fluorescen*s, *P. fulva*, *P. putida*, and *Pseudomonas spp.*	maize	improve the plant growth and productivity by abolishing the growth of toxic plant pathogens	(83−85)
cold-tolerant *Pseudomonas fluorescens* strain	pea	good plant growth enhancer and biocontrol agent	(86)
*Pseudomonas* strain *Pseudomonas aeruginosa*	tomato	positively modulated sugar production, improved the sweetness of tomatoes, and significantly enhanced fruit yield	(87,88)
*P. putida* and *P. fluorescens*	*Hyoscyamus niger*	reduced the damaging effects of water deficit stress and increased the root length and no. of leaves	(89)
*P. putida*	tomato	improves growth and yield of fresh and dried tomato fruits under seawater irrigation stress	(90)
*P. nitroreducens*	*Arabidopsis thaliana, Lactuca sativa*	exhibited remarkable improvement in growth, particularly in regard to biomass	(91)
*P. putida* and *P. fluorescens*	*Oryza sariva*	increase the grain iron content to combat with the problem of iron shortage in rice crop	(92)
*P. fluorescens*	*Rubus* (blackberries)	improves plant yield, fruit quality, and flowering buds	(93)
*P. fluorescens* and *P. putida*	pepper and spinach	increase length of the plants and also improve vegetable quality	(94)
*P. fluorescencs* and *P. putida*	turmeric	increased the plant biomass, stem length, no. of leaves, and curcumin contents	(95)
*Pseudomonas* spp.	soybean and wheat	enhanced soil enzyme and nutrient uptake activity, also increased grain size and total plant yield	(96−98)
*P. putida*	artichoke	promotes phosphate solubilization	(99)
*P. fluorescens*	rice	shows plant growth promotional activity in rice crop under both in vitro and in vivo conditions	(100)
*P. fluorescens*	mustard	augmented overall plant growth and yield and reduced the symptoms of stem blight disease in mustard plant	(101)
*Pseudomonas* spp.	onion	stimulate growth and increase the mineral concentration of the onion	(102)
*P. aeruginosa*	chilli	enhancement effect on all growth parameters	(103)
*Pseudomonas* spp.	chickpea	produced siderophores and IAA and improved plant growth, nodulation, chlorophyll content, and leghemoglobin	(104)
*Azospirillum* and *P. fluorescens*	wheat	promoted the elongation of roots in crops	(105)
*P*. *chlororaphis*	tobacco	growth-promoting effect and has also been described to develop systemic resistance	(106)

## 5 Direct Mechanisms

### 5.1 Biological Nitrogen Fixation

The total nitrogen present in the atmosphere is 78%, but still it is not available to plants. One of the most efficient and ecofriendly ways to increase crop plant growth and productivity has proven to be the employment of N_2_-fixing microorganisms as biofertilizers. For sustainable agriculture and high yield, the use of biological nitrogen fixation is the best alternative to chemical fertilizers. Rhizobacteria are useful for fixing the nitrogen and make it obtainable to the plants.^107^ However, an enzyme nitrogenase is used by PGPR to convert nitrogen into ammonia, which is the usable form for the plants.^108^ The symbiotic bacteria, for example, rhizobia, and nonsymbiotic bacteria, for example, *Azocarus* (*Anabaena*, *Nostoc*), *Gluconacetobacter diazotrophicus*, *Azotobacter*, *Azospirillum*, *cyanobacteria*, etc., fix the atmospheric nitrogen into an available form.^109^

In 1955, Anderson reported the nitrogen fixation by the genus *Pseudomonas*.^110^ The structure of the gene that produced the nitrogenase enzyme and the optimal condition at which it is useful for nitrogen fixation were investigated using *P. stutzeri*, which was isolated from Chinese rice fields.^111^*P. fluorescens*, as well as fluorescent pseudomonads, were reported to stimulate nodulation in a chickpea plant.^112^

### 5.2 Solubilization of P

Phosphorus is the most important macronutrient after N for the growth and development of plants. A higher amount of P is present in an insoluble form in the soil but not available to plants. Monobasic and dibasic are the only forms in which P is available to plants.^108^ P-based fertilizers are frequently used to overcome the deficiency of P and to enhance the crop production.^113^ Microbes in the rhizosphere that convert the insoluble forms of P into available forms are usually known as orthophosphates. The use of these P-solubilizing bacteria (PSB) plays significant role in making P available to the plants. The P-solubilizing bacteria can dissolve the insoluble P by producing organic acids with low molecular weights. Several bacteria produced citric and gluconic acid.^153^ Oteino reported that bacterial genera including *Bacillus*, *Enterobacter*, *Rhodococcus*, *Mesorhizobium*, *Flavobacterium, Rhizobium*, *Pseudomonas*, *Erwinia*, *Burkholderia*, *Microbacterium*, *Beijerinckia*, *Serratia*, and *Arthrobacter* can solubilize phosphates.^114^

### 5.3 Production of HCN

HCN is a volatile compound that has antibacterial^115^ as well as antifungal characteristics.^116^ HCN is a very toxic compound used against phytopathogens. To inhibit phytopathogens and increase yield, most of the PGPR produced HCN.^117^ HCN production is the trait of *P. aeruginosa* that is already well-known in disease inhibition.^118^ A secondary metabolite, HCN produced by *Pseudomonas entomophila*, and entomopathogenic bacteria, has been linked to biological control and pathogenesis in many other bacteria.^119^ In a recent study, HCN-producing fluorescent *Pseudomonas* and non-HCN-producing *Bacillus licheniformis* in a mixed culture were reported to improve the vegetative growth and photosynthetic parameters in a wheat crop.^120^

### 5.4 Production of Siderophore

Iron is found in its native state as ferric ions and Fe^+3^, which are slightly soluble; therefore, these forms are used neither by plants nor by microorganisms. Some extracellular compounds have been identified to be secreted by PGPR, usually known as siderophores. Kumar and co-researchers reported that siderophores are small iron-containing molecules with a high affinity for chelating chemicals released by the plant and microorganisms, acting as highly soluble Fe^3+^-binding chelating agents.^113^ David et al. reported that siderophores producing *Pseudomonas* spp. are important for boosting plant growth and combating a variety of plant diseases.^121^ Because of its function in both biological disease control and plant pathogen virulence, siderophore formation by plant-associated microbes is of much interest. The production of siderophores that takes place by bacteria like *Pseudomonas* produces pyoveridins; *Agrobacterium tumefaciens*, *Erwinia chrysanthemi*, and *Enterobacteriacea* generate catechols; *Erwinia carotovora*, *Enterobacter cloacae*, and other fungi make hydroxamates; and *Rhizobium meliloti* produces rhizobactin.^122^

### 5.5 Phytohormone Production

Phytohormones are very essential for plant development. Most important hormones are gibberellins, indole-3-acetic acid (IAA), and cytokinins. The most prevalent and well-studied auxin, IAA, has a crucial role in the differentiation and development of tubers and seeds, as well as cell division. It also controls vegetative development and initiates the production of the lateral and adventitious root.^123^ Auxin is produced by different bacterial genera like *Pseudomonas*, *Xanthomonas*, and *Rhizobium*, as well as *Bacillus cereus*, *Enterobacter cloacae*, *Bradyrhizobium japonicum*, *Serratia marcescens*, *Burkholderia*, *Azotobacter*, *Alcaligenes faecalis*, and *Mycobacterium* sp., etc., which help in plant growth and development. Similar to this, cytokinins play an essential role in cell enlargement, cell expansion, and cell division.^124^ Gibberellin changes the morphology of plants, especially in stem tissues, and extension of cells takes place. Ethylene hormone is in gaseous form and is usually known as a wounding hormone because any physical or chemical perturbation is the reason for its production. The root growth is inhibited by the production of ethylene. Therefore, PGPR play an important in reducing ethylene production by mediating the phytohormones, maintaining ion homeostasis, and regulating the expression of stress-responsive genes.^125^

### 5.6 Production of Antibiotics

**Figure 2:**
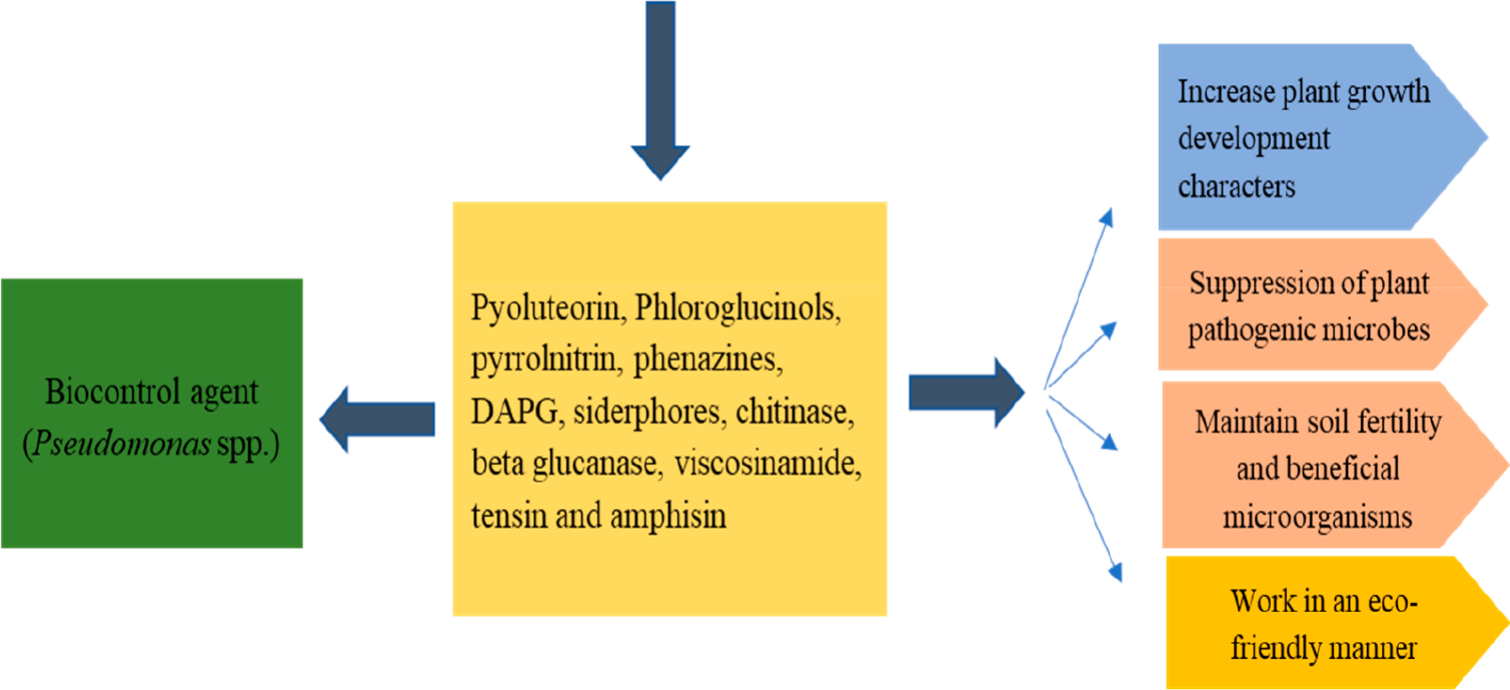
Antibiotics production by *Pseudomonas* spp. and their impacts on plants.

Many distinct types of microbes produce toxic compounds that are hazardous to other pathogen microbes. However, it is advantageous to plant growth and development. These are antibacterial or microbial toxins that can be harmful to or eliminate other bacteria even in low quantities.^126^ Kumar et al. evaluated that DAPG acts as an antibiotic toward fungus, bacteria, helminths, and other parasites because *Pseudomonas* spp. synthesize pyrrolnitrinis.^123^*Pseudomonas* spp. produce DAPG, which is involved in the inhibition of phytopathogens. A strong antibiotic phenazine of *P. fluorescens* has been utilized to combat all *G. gramini* infections of wheat.^127^ Fluorescent *Pseudomonas* produces antibiotics and acts as a biocontrol agent.^157^ Pioluteorine, phenazine-1-carboxylic acid 2,4-diacetylphloroglucinol (Phl), and pyocyanin are some of the potential antibiotics synthesized by *P. fluorescens*.^128^ These antibiotics increase plant growth, suppress phytopathogens, and also enhance the soil fertility (Figure 2).

## 6 Indirect Mechanisms

### 6.1 Antifungal Activity

PGPR stimulate the plant development, which prevents the spread of phytopathogens.^129^ Bacteria produce antifungal antibiotics, which help in the growth inhibition of pathogenic fungi. For example, *P. fluorescens* produces 2, 4-diacetylphloroglucinol. Some PGPR breakdown the fusaric acid produced by the *Fusarium* spp. associated with wilt disease and therefore prevent the pathogenesis. *Pseudomonas stutzeri* produces enzymes that can break down the cells of *Fusarium solani*. Recently, *P. fluorescens* was suggested as biocontrol agents because they protect plants against several fungal diseases, like black root-rot of tobacco, root-rot of wheat, and root-rot of a pea, etc. In a recent study, *P. aeruginosa* was characterized to produce certain metabolites, bioactive compounds, and antifungal compounds like 2,4-diacetylphloroglucinol (2,4-DAPG), pyoluteorin, and pyrrolnitrin that make this bacteria effective against a broad range of phytopathogenic fungal agents.^130^ Similarly, *P. stutzeri* isolated from *Withania somnifera* seeds was found effective in the suppression of the mycelial growth of *F. oxysporum* var. *ciceri* and *R. solani*.^73^

### 6.2 Induced Systemic Resistance (ISR)

In the rhizosphere of plants, beneficial microbes induce systematic resistance as a defense in response to pathogenic attacks.^131^ Microbes assist in structural changes that promote defense mechanisms via the accumulation of biochemical and phenolic compounds.^132^ In a study, decreased susceptibility to wilt disease in carnation plants and foliar disease in cucumber due to the induction of systematic resistance was observed.^133^*Pseudomonas* strains also assist in inducing systematic resistance. *Pseudomonas* spp. like *P. putida* and *P. fluorescens* protect against pathogens in plants like tomato, sugar cane, and oak.^134^ ISR induction on plants by PGPR result in substantial modifications in the plants structure and functions that lead to resistance against invading pathogens.^135^

### 6.3 Rhizoremediation and Stress Control

**Figure 3:**
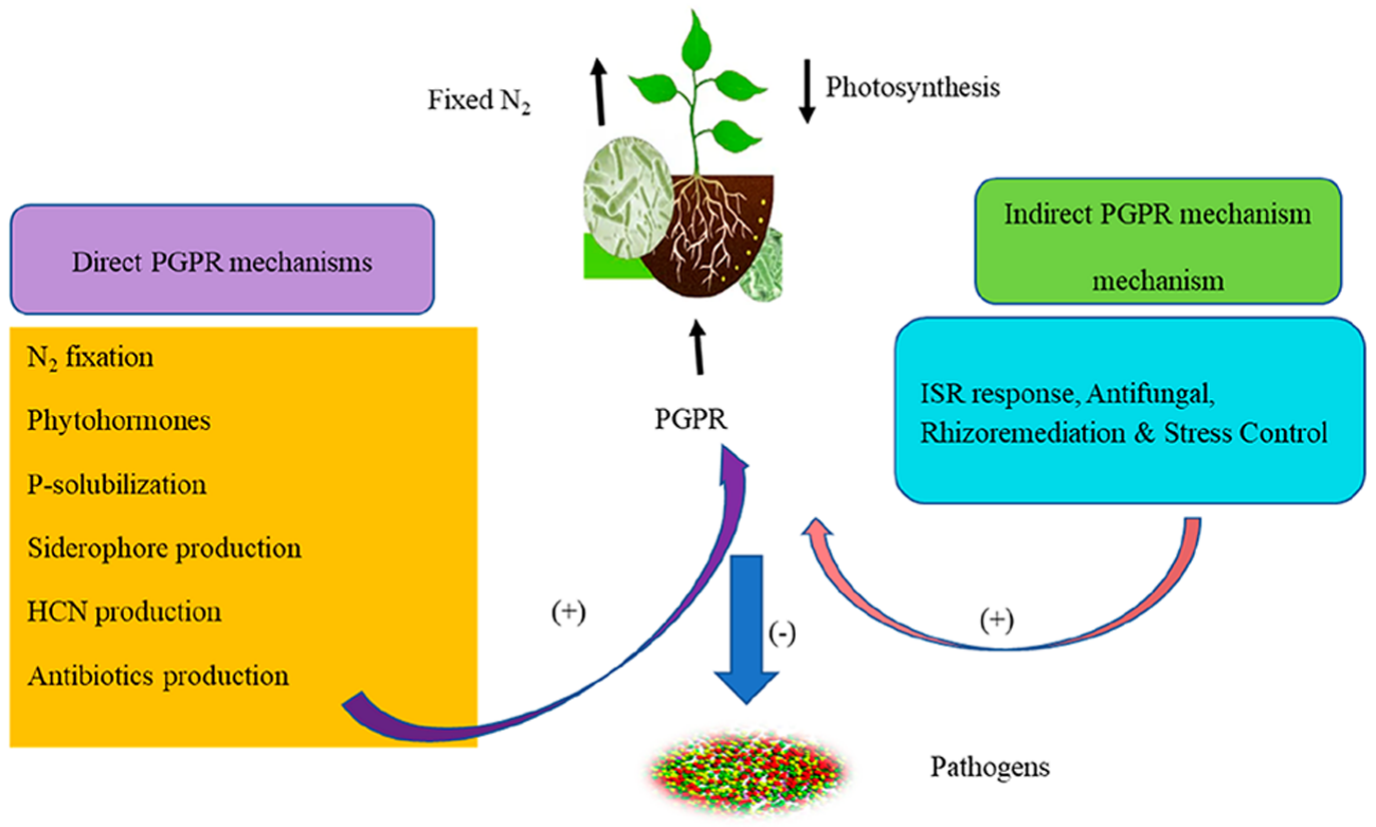
Direct and indirect roles of *Pseudomonas* spp. in growth promotion and disease suppression.

Islam and co-researchers discussed that plants are frequently exposed to several stresses both abiotic and biotic that ultimately have an impact on plant development and survival.^136^ Plants and microbes are potentially used in phytoremediation to eliminate harmful metals efficiently and economically from the polluted environment.^137^ Heavy metals (HMs) are harmful to plant growth^138^ and establishment, and certain microbes detoxify them. When the microbes are added to the contaminated soils, they lower the deleterious effects of metals on plants growth. A recent study has proved that the inoculation of *Sedum alfredii* with *P. fluorescens* improved the net photosynthetic rates, intercellular CO_2_ concentration, transpiration rate, and stomatal conductance and upregulated the photosynthetic genes, which promote both the growth and Cd uptake ability of the plant.^139^ Similarly, application of copper-tolerant siderophore- and ammonia-producing *P. lurida* on *Helianthus annuus* significantly improved the growth and phytoremediation ability of plants grown under Cu contaminated soil.^140^*Pseudomonas* spp. are also reported to enhance plant growth and improve the ability of the plants to survive under drought conditions,^141^ heat shock,^142^ and high salinity.^143^ The direct and indirect modes of action of PGPR is presented in Figure 3.

### 6.4 Role of Pseudomonas Species in Plant Disease Management

According to current estimates, a significant amount of food products and crops are lost each year due to various diseases caused by pathogens. These plant diseases are the major factor affecting food production and human societal development.^144^ Generally, cereal crops are more commonly affected by soil-borne diseases. For plant disease control, the application of biological control agents has been recognized as an effective method.^145^ The efficient control of diseases with the help of PGPR has been testified in many crop plants.^146^ PGPR as biocontrol agents are reported to have a remarkable ability to increase crop production and tolerate abiotic and biotic stresses.^147^

The term biocontrol is often employed for managing plant diseases that occur during the storage of food and at various plant growth stages. The studies on biocontrol by rhizobacteria are usually focused on controlling many pathogenic microbes.^148^ Numerous bacterial isolates are known that act as biocontrol agents. Among several biocontrol agent, *Pseudomonas* spp. have been broadly studied as more dominant bacteria possessing the potential to defend plants against various notorious plant pathogens (Table 2)^149^ and promote plant growth under stress conditions.^150^*Pseudomonas* bacteria has ability to lessen the disease occurrence by developing inducible plant defense mechanisms, which make plants more resistant against invading pathogens.^151^ Bacterial strains prevent pathogenic growth using various mechanisms such as by secreting the antibiotics and HCN; producing cell-wall-degrading enzymes like β-1,3-glucanase, chitinase, toxins, biosurfactants, and important metabolites; and by competing for minerals.^152^ The earliest known mechanism of biocontrol includes the production of beneficial compounds like siderophores, which proficiently sequester Fe (iron) and kill pathogens.^153^ Disease control properties are also associated with various secondary metabolites coded by genes present in cores and flexible parts of the bacterial genome.^154^ Through genome sequencing, it is predicted that approximately 6% of the genome of *P. fluorescens* Pf-5 secretes secondary metabolites, which indicates the direct link between secondary metabolite production and genetic elements^155^

**Table 2:** List of *Pseudomonas* Strains That Take Part in Disease Management

bacterial strain	plants	role of bacterial strain in disease management	references
*P. fluorescens*	tomato	significantly inhibited the virulence traits of *Ralstonia solanacearum* that cause tomato wilt disease	(164)
*P. putida* and *P. aeruginosa*	rice	these isolates effectively reduced the disease incidence of sheath blight and developed resistance to *Rhizoctonia solani*	(165)
*P. aeruginosa*	cowpea	increase plant productivity and reduce the symptoms of blight disease in cowpea caused by bacteria (*Xanthomonas campestris*)	(166)
*P. fluorescens*	tobacco	reduce the growth of tobacco mosaic virus by making an antibiotic protein	(167)
*P. fluorescens* (Pf1 and TDK1)	ground nut	shows efficacy against the collar rot pathogen and leafminer insect	(168)
*P. fluorescens*	mung bean	has biocontrol potential against the root rotting of fungi and also promotes plant growth	(169)
*Pseudomonas*	cucumber	combated cucumber damping off disease and used in integrated disease control	(170)
*P. fluorescens* strain	turmeric	directly or indirectly involved in disease resistance in turmeric against turmeric rhizome rot	(171)
*P. fluorescens* M, *P. putida* L, and *P. aeruginosa* J, B	apple orchards	lessen the symptoms of apple replant disease and ensure the good health of plants	(172)
*P. fluorescens*	cabbage	combating black rot of cabbage	(173)
*P. aeruginosa*	chilli pepper	managing anthracnose in chilli pepper by fighting against *Colletotrichum truncatum* and *Colletotrichum capsici*	(174−176)
*P. fluorescens*, *P. putida* and *P. cepacia*	tomato	management of tomato early blight pathogen (*Alternaria solani*)	(177)
*Pseudomonas* strains	cucurbit	controlling the powdery mildew disease by developing systemic resistance in the host plant	(178)
*P. fluorescens*	tomato	very effective in stopping the infection of tomato (*Lycopersicon esculentum*) leaves by *P. infestans* and notably reduced the expansion of late blight lesions.	(179)
*P. fluorescens*	maize	control corn sheath blight by inhibiting the growth of *Rhizoctonia solani*	(180)
*P. fluorescens* Pf1 and Py15	mulberry plant	protect the mulberry plant from root rot disease induced by *Macrophomina phaseolina*	(181)
*P. aeruginosa*, *P. syringae*, and *P. fluorescens*	tomato	rested species reduced the incidence of tomato bacterial wilt caused by *Ralstonia solanacearum* and promoted the growth of the tested tomato genotypes	(182)
*P. protegens*, *P. donghuensis*, and *P. aurantiaca*	cotton	control *Verticillium* wilt disease by producing effective antipathogenic metabolites against *Verticillium dahlia*	(52, 183, 184)
*P. chlororaphis*	wheat	antimicrobial properties against Gram-positive bacteria and actinomycetes	(185)
*P. koreensis* MG209738 and *B. subtilis* MF497446	maize	have an inhibitory effect on late wilt disease caused by *Cephalosporium maydis* in the maize plant and lead to plant growth promotion	(186)
*P. putida*	corn	increase in shoot and root weight of corn plants inoculated with the strain of *P. putida* that is famous due to its anti-fungal properties	(187)
*Pseudomonas*	annual bluegrass, bermuda grass, and rice	produce growth inhibition compounds that prevent the *Fusarium* pathogen infection	(188)
*Pseudomonas* spp.	pine seedlings	decrease the symptoms of pine wilt sickness induced by pine wooden nematode (*Bursaphelenchus xylophilus*)	(189)

The biocontrol ability of *Pseudomonas* spp. has been reported in some important plants (tomato, wheat, and chickpea).^156^ Rhizobacteria produce a variety of secondary metabolites, antibiotics, and bioactive compounds that make them excellent biocontrol agents. A research study has reported the antimicrobial traits of a siderophore-producing *Pseudomonas* strain against *Rhizoctonia solani* and *Sclerotium rolfsii*.^157^*P. fluorescens* produced 2,4-DAPG, which controls and manages take-all disease in *Triticum aestivum*.^158^ Orozco-Mosqueda et al. showed that the trehalose production by the *Pseudomonas* UW4 played a significantly role in protecting tomato plants against salt stress.^159^ Hydrogen cyanide-producing *P. chlororaphis* exhibits nematocidal activity against *Meloidogyne hapla* nematodes by decreasing the number of galls in tomato plants and by killing juveniles both in vitro and inside the plants.^160^ A *P. putida* strain was revealed to decrease the damage instigated by *Pythium ultimum* on tomatoes and also increase the growth of the infected plants^161,199^. Many other recent studies have highlighted the beneficial impacts of *Pseudomonas* spp. on improving the plant growth by suppressing the colonization of phytopathogens.^43,162,163^

## 7 Production and Effects of Bacterial Volatiles

Low-molecular-weight volatile organic substances promote plant development.^190^ The importance of volatile organic compounds (VOCs) in inducing resistance in host plants is comparable to the function they play in insect resistance.^43^ Microorganisms interact with plants in distinctive ways.^191^ Volatile natural compounds of microbial origin possess antimicrobial properties, promote plant growth, and serve as alerts by producing a variety of signaling molecules.^192^

Most research on the consequences of bacterial fluctuations on flora have been accomplished thought the use of a bifurcated Petri dish, where in the two compartments are separated through a plastic rim. This permits the exchange of volatile compounds while also preventing the spread of nonvolatile metabolites via the medium. In addition to CO_2_, generation that probably contributes fairly to growth promotion was discovered in closed Petri dishes.^193^ The use of *P. chlororaphis* showed that butanediol no longer only promotes plant development but also induces systemic resistance and drought tolerance^194^

Intriguingly, beneficial microbes are well-known to produce a variety of dynamic volatiles that protect the plants from phytopathogens, and gas chromatography–mass spectrometry (GC-MS) has helped in profiling the microbial volatile organic compounds (VOCs). A recent study has highlighted the potential role of microbial volatile organic compounds (VOCs) produced by endophytic *P. putida* associated with black pepper in suppressing the mycelial growth of various fungal pathogens.^195^ Similar to this, the volatile profile of *P. chlororaphis* subsp. *Aureofaciens* revealed that 3-methyl-1-butanol, phenylethyl alcohol, and 2-methyl-1-butanol were the most abundant VOCs, which showed strong inhibition toward *C. fimbriata* associated with black rot disease in sweet potato.^196^ SEM analysis has revealed that VOCs affect the morphological and structural features of *B. cinerea* and thus suppress fungal growth.^197^

## 8 Regulation of Secondary Metabolism and Biocontrol

The two-component system GacS/GacA in *Pseudomonads* is essential for controlling how biological control components are expressed in a variety of pseudomonads.^38^ The GacS sensor kinase is activated by unknown inputs, and the phosphorus relay then activates the GacA response regulator. When cells attain large population densities, positively activated GacA regulates the transcription of short regulatory RNAs. These short ribonucleic acids attach to distinct proteins, RsmA and RsmE, and by activating specific genes they relieve translational repression. The GacS/GacA system regulates 241 genes in *P. aeruginosa*, with nearly whole overlap with genes under the control of small regulatory RNA.^198^

A competitive nonpathogenic root colonizer, *P. chlororaphis* strain O6, served as another example of the GacS/GacA signaling pathway’s regulatory strength. As advantageous features, the O6 strain generates several secondary metabolites, like phenazines, formonitrile (HCN), siderophore, and pyrrolnitrin. Abiotic stresses including drought and salt as well as *P. chlororaphis* induced systemic resistance in roots through colonization,^199^ the induction of triggered systemic resistance^200^ via the upregulation of the volatile 2,3 butanediol,^160^ and the expression of RpoS and, therefore, different genes structured in this opportunity σ-factor. A recent past study has revealed the pathways through which beneficial bacterial metabolites confer protection against pests and pathogens. During the root colonization, metabolites are produced that are controlled by the global regulatory Gac/Rsm signaling cascade, which is linked with the other regulatory pathways. The Gac/Rsm regulation aids in expressing the genes that encode these beneficial traits in *P. chlororaphis*.^201^ It is interesting to note that 4-carbamoyl acetic acid, another systemic resistance inducer in plants, is not always under GacS regulation.^106^

Proteomic analyzes of the characteristic of regulatory mutants were used to find out which pathways are coregulated. Recent studies on GacS variants revealed new proteins with as yet poorly defined functions in bioregulation. One of these proteins, PspB, is a serine protease that is connected to the DNA repair proteins that frame shifting and aggregation (a putative single-strand binding protein, Ssb, and recombination-related protein, RdgC); isoprenoid production including synthetic proteins, GATase1 ES1, and glucose 1 thymidylyl phosphate transferase; RmlA, probably concerned in hair-related proteins; polyketide metabolism; and cell membrane organization (CpaC and LysM mandatory peptidoglycans), also in outer membrane protein (OprF).^202^ These findings have highlighted the intricacy of the regulons involved in the appearance of the biological regulation phenotypes within a single agent. Other important regulators are also expected to exist in *Pseudomonads* spp. and other bacteria with efficient biological control traits.^203^

## 9 Plant Metabolism Interaction Induces Resistance and Priming

Plants depend on immunogenicity to defend themselves against pathogenic attack. This acquired immunity completely depends on and encourages protective reactions. Preformed defenses are nonspecific and include structural barriers like the cell wall and cytoskeleton that keep viruses and parasites at bay, as well as chemicals with effects.^204^ The pathogen’s surface pathogen-associated molecular patterns (PAMPs) or proteins (effectors) that the pathogen has translocated inside the host cell are recognized to activate the induced defenses.^205^ For example, in the case of microbial infection, plant immune response in the form of hypersensitive cell death is an important event to prevent the spread of attacking pathogens,^206^ the production of free radicals,^207^ plasma membrane defenses,^208^ and phytoalexin synthesis.^209^ On the other hand, the next opportunity in the protection response process is the transcription of pathogenesis response proteins^210^ and a kind of planned cell death (also known as hypersensitivity reaction (HR), which restricts the microbial ability to spread).^211^

For a long time, it is been counseled that the function of basic metabolism for the duration of plant pathogen relation is to aid the cell energy demand for plants. Because of the amount of gene production from various defense pathways, energy is required when carrying out the plant protection responses.^212^ Additionally, defensive reactions seem to manipulate health value because, like in *Arabidopsis*, plants that integrate specific protection reactions are undersized and feature decreased fertility compared to mutant plants with deficient protective signaling pathways, which have higher fertility.^213^ Anyhow, it seems that in order to create a strength level that is favorable to defense, the up-regulation of the associated protection pathways is compensated with the aid of using the down-regulation of the genes involved in different defense-associated pathways. Supporting this idea was the fact that genes concerned in photosynthesis and chlorophyll production are down -regulated in response to pathogen-derived elicitors, both pathogenic and nonpathogenic infections, and both virulent and avirulent infections.^214^

Utilizing fluorescence imaging of chlorophyll in special interactions between plant microbes, changes in photosynthesis have been reported regionally at the site of infection and in surrounding tissues and decreases in photosynthesis became quicker and greater after inoculation with an a virulent strain.^215^ However, because light responses all through photosynthesis produce chloroplast ROS that may be utilized for protection responses, the overregulation of photosynthesis is unreasonable.^216^ There is no experimental proof to explain why it occurs. However, two potential pathways have been put forward: (1) photosynthesis suppression brought on by pathogenic effectors^217^ and (2) sugar signal moderation causes regulation.

In any way of the mechanism, it is likely that down-regulating photosynthesis reduces the amount of energy required when other mechanisms that provide this energy are up-regulated. For instance, boosting the activity of cell wall invertase, respiratory metabolism, and glucose transporters can produce energy.^218^ Formation of plant secondary metabolites like phytoalexins and the expression of protection-associated genes can both be further enhanced by this metabolic switch from supply to sink.^219^ Protection tactics for dangerous surroundings have a reasonable cost. The epigenetic law of priming could permit plants to shield their offspring from recurrent biotic stress instead of undergoing costly irreversible genetic fixation of the trait and its associated costs. More information on this process will open possibilities to enhance sickness resistance in agricultural crops. In order to protect crops and reduce the use of pesticides and other harmful chemicals to treat diseases, it is vital to create sustainable methods of pest and disease control in the twenty-first century.^220^

## 10 Lytic Enzymes and Plant Disease Control

The one of the best biocontrol mechanisms used by *Pseudomonas* spp. to eradicate soil-borne pathogens is the formation of cell-wall-degrading enzymes (Table 3).^221^ Hydrolytic enzymes that break down the cell wall include of β-1,3-glucanase, protease, cellulose, and chitinase produced by biostimulant strains of PGPR, and they directly suppress the pathogens proliferation.^222^ Chitinase and β-1,3-glucanase activity have the potential to substantially destroy the chitin chain, which is an insoluble linear polymer of α-1, 4-N-acetylglucosamine.^223^

**Table 3:** Role of Lytic Enzymes in Plant Disease Control

bacteria	plant	disease management by lytic enzymes	references
*P. stutzeri*	ashwagandha	Secrete Chitinase, protease and lipase enzymes against *Fusarium oxysporum* and *Rhizoctonia solani*	(73)
*P. putida*	tomato	Enzymes of the lipoxygenase pathway are very effective against *Botrytis cinerea*	(151)
*P. fluorescence*	maize	produce amylase that causes a reduction in both root and foliar diseases during the maize growing season	(233)
*P. chlororaphis*	canola plants	formed chitinase and β-1,3-glucanase and used to control *Sclerotinia* stem rot of canola	(11,234)
*P. putida*	bean	secrete lipase, protease, and chitinase effective against common bean rust disease caused by *Uromyces appendiculatus*	(235)
*P. fluorescens*	tomato	suppression of wilt disease of tomato caused by *Ralstonia solanacearum* by secreting the many enzymes, especially peroxidase, lipoxygenase, polyphenol oxidase, phenylalanine ammonia lyase, etc.	(236)
*P. aeruginosa*	sugar cane	secrete hydrolases and oxidoreductases enzymes to control sugar-smut disease	(62)
*P. aeruginosa*	chilli	release polyphenol oxidase, phenylalanine ammonia lyase, amylase, per-oxidase etc. these enzymes eradicate the chilli anthracnose infection by inducing systemic resistance in chilli	(176)
*Pseudomonas* spp. *P. fluorescens*	cucumber	combat the cucumberroot rot and *Pythium* damping-off disease by producing β-1,3-glucanase and protease. these enzymes have an antagonistic property	(237)
*P. fluorescens*	safflower	liberate peroxidase, PAL, and β-1,3-glucanase used against *M*. *phaseolina* root rot of safflower disease	(238)
*P. aeruginosa*	peanut	production of the chitinase enzymes triggered the lysis, perforation, and fragmentation of the hyphae of pathogen *S. sclerotiorum*, which cause stem rot of peanut	(239)

Within the *Pseudomonas* genus, certain species secrete β-1,3 glucanases that contribute to the destruction of many pathogens: in *P. cepacia*, the β-1,3 glucanase reduces the disease incited by the toxic pathogens, e.g., *S. rolfsii*, *P. ultimum*, and *R. solani*.^223^*Pseudomonas*strain K32 is HM-resistant, and its biological control ability is verified against six *Oryza sativa* fungal pathogens: *Paecilomyce* ssp., *A. parasiticus*, *A. flavus6Cladosporium herbarum6Alternaria alternate,* and *Rhizopus stolonifer*. The inhibitory activity was ascribed to the production of β-1,3-glucanase, chitinase, and protease enzymes.^224^ Apart from showing the formation of β-glucanases and chitinase enzymes, *Pseudomonas* spp. obstruct the proliferation of *R. solani* and *P. capsici*, which are the most dangerous crop pathogens across the globe.^225^ In *P. chlororaphis*, quorum sensing (QS) regulates the exoenzyme production, antifungal substances, and biofilm formation.^226^ QS is a cell-to-cell communication system that depends on cell density that regulates bacterial gene expression through signaling molecules (autoinducers), such as biofilm formation, bacterial virulence, and pigment production^227^

Saritha et al. showed that *P. putida* discharges various important substances such as siderophores, hydrogen cyanide (HCN), chitinase, protease, urease and ACC-deaminase that impede mycelial growth of *S. rolfsii6F. oxysporum*, and *C. fimbriata* and as a result ensure the plant’s good health.^228^*Pseudomonas fluorescens* strains also have aminocyclopropane-1-carboxylic acid- or ACC-deaminase activity^229^ that significantly controls the quantity of plant ACC-deaminase left behind for the biosynthesis of ethylene.^128^ Moreover, ACC-deaminase decreases undesirable effects of the stress brought by ethylene on plants by diminishing its quantity,^230^ although it has been noted that a rapid decrease in ethylene levels can reduce seed emergence.^231^ The expression of ACC deaminase by the endophyte, *P. migulae* 8R6 can make *Madagascar periwinkle* more resistant to phytoplasma infection.^232^

## 11 *Pseudomonas* Species Producing Rhamnolipids (Biosurfactants)

The rhamnolipids (RLs) are a special class of biosurfactants, and quorum sensing (QS) molecules regulate their secretion. Surfactants are very significant in QS molecules or cell-to-cell interaction, cellular differentiation, bacterial cell movement, and making of water canals, all which are properties of the *Pseudomonas* genus. These natural substances are secreted by the microbial cells^240^ and have numerous benefits as compared to synthetic surfactants, having very little toxicity, excellent ecofriendly properties, biodegradability, acceptable surface activity at high pH, temperatures, and salinity levels, and the capability to be produced from the renewable feedstock.^241^ They are extensively used in pharmaceuticals, agriculture, pesticide removal, the food industry, household cleaning, and improvement in oil recovery.^242^ In agriculture, RLs effectively take part in disease management by diminishing the growth of phytopathogens. These biosurfactants also have antiviral, antifungal, and antibacterial potential.^243^ The benefits and market potential of RLs have been extensively studied,^244,245^ e.g., the virucidal effects of RLs were recalled when COVID-19 emerged as global issue.^246^ RLs are also employed in the removal of HMs like As, Cr, Pb, Cd, and Ni from soils due to their anionic character and make the soil stress-free.^247^

RLs are primarily formed by *P. aeruginosa*, although their secretion by some other *Pseudomonas* species has also been reported.^247^*P. aeruginosa* produces the majority of the glycolipid-type rhamnolipids, which are the most extensively studied biosurfactants.^248,249^ This is due to their good surface activities, high yields after relatively shorter incubation periods, and ease microbe culviation. However, in *P. aeruginosa*, three QS systems exist including las, rhl, and pqs systems, which are responsible for regulating the RLs production.^250,251^ The *P. aeruginosa* rhamnolipids illustrates a vast variety of the microbes such as *Micrococcus luteus*, *Escherichia coli*, *S. epidermidis*, *A. faecalis*, *Mycobacterium phlei*, and *Serratia marcescens*.^252^ Moreover, *P. aeruginosa* RLs also exhibited antifungal potential against *Penicillium chrysogenum*, *Aureobasidium pullulans*, *Aspergillus niger*, and *Chaetomium globosum*. Similarly, RLs produced by a *P. aeruginosa* strain (DS9) were reported to exhibit antifungal activity against *Fusarium sacchari* instigating Pokkahboeng disease.^253^ Similarly, in another research report, the antifungal property of RLs against seven plant pathogens was surveyed. The outcomes reveal the high ability of RLs derived from *P. aeruginosa* (ZJU211) to fight three Ascomycota, two Oomycetes, and two Mucor species.^254^ The very first report on the insecticidal activity of RLs was reported by Kim.^255^ They described that the rhamnolipid formed by *Pseudomonas* strain EP-3 has disruption ability against *Myzus persicae* (green peach aphid). The biofilm resistance against antimicrobial agents is becoming a worldwide problem,^256^ whereas RLPs have prohibiting effect on the biofilm formation.^257^

## 12 Plant Protection against Abiotic Stress

PGPR belonging to various taxonomic groups colonize the plant rhizosphere and mediate stress responses by employing multiple mechanisms. These beneficial becteria facilitate the acquisition of essential nutrients or modulate the production of plant hormones and thus develop in plants the ability to withstand the abiotic stresses.^48^ In addition to promoting tomato plant development in the absence of stress conditions, the endophytes *P. brassicacearum* exhibit YsS6 and *P. migulae* 8R6, both of which display ACC deaminase activity, and also caused biomass accumulation to be considerably colder, drier, and higher in chlorophyll and more blooms and shoots in tomato vegetation grown under treated under salt stress conditions.^258^

Liu and his co-workers recently discovered that inoculating a tomato plant with ACC deaminase-producing P. *azotoformans* CHB 1107 improved the dry weight of the plant shoots and roots and significantly decreased the plant’s reaction to salt stress by emitting less ethylene. These advantageous outcomes disappeared as *P. azotoformans* CHB 1107 M was used to vaccinate plants (acdS variant). Furthermore, it considerably improved plant uptake of potassium, manganese, and calcium in contrast to the *P. azotoformans* CHB 1107 acdS variant.^82^

The ability of *Pseudomonas* spp. to express ACC deaminase and decrease the ethylene concentration in plants is associated with its capacity to minimize heavy metal quantity in certain plant tissues.^259^*P. seudomonas* also exhibited greater resistance to other abiotic stressors when foreign ACC deaminase genes were expressed. Similar to this, inoculation of freeze-stressed tomato plants with *P. frederiksbergensis* OS211-acdS (carries the ACC deaminase and pRKACC plasmids) lowered ethylene emissions, reduced the ACC quantity, and decreased ACC oxidase activity. *P. frederiksbergensis* promoted plant development more than the uncultivated strain without ACC deaminase,^260^ evidencing the useful impact of ACC deaminase expression.

HM pollutants, salinity, drought, and low/excessive temperature are vital climate stressors that have an effect on regular plant physiological and biochemical processes, thereby significantly lowering plant development and productivity. The creation of numerous plant-useful metabolites may also amplify the impacts of environmental stress in plants, as well as to the useful activities of PBPs in plant development and biological manipulate activity. The sections that follow focus on the advantageous mechanisms of connection among PBPs and plants and how these relationships promote plant development and improvement in response to various forms of environmental stress.^261^ Under drought conditions, the genes related to the synthesis of compatible solutes, exopolysaccharides, and secretion systems (type II, III, IV, and VI) were observed to be upregulated in *P. fluorescens*, which aided in alleviating plant stress.^141^ The potential of Pseudomonas spp. could be effectively used to enhance the plants’ ability to survive under abiotic stress conditions.

## 13 Commercial Products Based on *Pseudomonas* spp.

With the rapidly increasing interest in ecofriendly biocontrol of soil-borne plant pathogens, many companies currently have developed biological control agents, biofertilizers, and biofungicides as commercial products under several trade names (Table.4). Several reports have explained the potential applications of bacterial inoculants for the maintenance of natural agriculture, for instance, biofertilization, biostimulant, phytostimulation, and bioremediation.^262^ The basic aim of PGPR-based biocontrol products is to support sustainable approaches for the plant disease management. The two biggest potential markets for biocontrol products are now Europe and the United States, with South America coming in third.^263^ Many rhizobacteria have been scrutinized at the laboratory level, and a few of them are successfully commercialized globally.^264,265^

**Table 4:** List of Commercially Available *Pseudomonas* spp.-Based Biocontrol Products^a^

biocontrol species	product name	application	plants	target pathogen species	manufacturing company
*Pseudomonas syringae* ESC-10 and ESC-11	Bio-Save 10LP, 110	lyophilized based products, frozen cell pellets added to H_2_O to make a homogeneous suspension of liquid. it is applied to postharvest fruit as a spray, drip, or drench process	potatoes, cherries, and pome fruit	*Geotrichum candidum*, *Mucor pyroformis*, *Penicillium* and *Botrytis cinerea*	EcoScience Corporation (Longwoord, FL)
*Pseudomonas aureofaciens* Tx-1	BioJect Spot-Less	applied liquid as spray droplets	turf	dollar spot	Eco Soil Systems Inc.
*Pseudomonas chlororaphis* strain	Cedemon	applied seed treatment as a seed dressing method	barley and oat plants	includes spot blotch, leaf spot, leaf stripe, net-blotch, and *Fusarium* spp.	BioAgri (Sweden and Uppsala)
*Pseudomonas fluorescens* A506	BlightBan A506 (EPA registered)	at bloom time, applied wettable powder as a spray	used blueberry, pear, almond, tomato, cherry, apple, apricot, peach, potato and strawberry	russet-inducing microbes and *Erwinia amylovora*	NuFarm Inc. (Burr Ridge, IL)
*Pseudomonas chlororaphis* 63–28	AtEze*	liquid applied as a drench	greenhouse grown vegetables and ornamental plants species	*Fusarium oxysporu*, *Rhizoctonia solani*, and *Pythium sp.*	Eco Soil Systems Inc. (San Diego, CA)
*Pseudomonas fluorescens* A506, 1629RS	FROSTBN*	applied early in the growing season liquid as spray	tomato, almond, potato, and some fruit plants	frost-forming microbes	Frost Technology Corporation
*P. syringae*	EPA registered 1992				Plant Health Technologies (CA and Lathrop)

a The Information about the products is adapted from a list generated by Dr. D. Fravel, USDA-ARS 2000 and updated by the APS Biological Control Committee (http://www.oardc.ohio-state.edu/apbscc).

The applications of *Pseudomonas* inoculants as PGPR in Europe need authorization under the appropriate directives. These significant directives have been reviewed by many researchers.^265,266^ Furthermore, plant protection products protect the plants against damage causing agents, have a direct impact on plants, and are used against undesirable plants such as herbicides. However, European legislatures have concerns with the usage and registration of plant protection commercial products that are addressed in Council Directive titled “The Plant Protection Directive”, and its implementation is under the power of EFSA (European Food Safety Authority). This gives a complete framework for generating an official inventory of compounds that cause no hazard to the ecosystem. These beneficial compounds are authorized for utilization and subsequently can be marketed in any part of the world. In the 1990s, numerous *Pseudomonas* strains were approved as registered biopesticide products by the U.S. Environmental Protection Agency (US EPA). These *Pseudomonas* spp. are enlisted on USEPA biopesticide Web site and discussed in detail^267^ (Table 4).

## 14 Conclusion

Rapidly growing plant pathogens and climate changes bring severe changes in food security and crops yield. In the present scenario, plant-growth-promoting rhizobacteria (PGPR) are some of the good suitable choices for the enhancement of plant growth and disease management over the consumption of chemical fertilizers and pesticides. Among many PGPR and biocontrol agents, *Pseudomonas* spp. play a crucial role in controlling the viability and crop-destruction activities of phytopathogens. Their biocontrol activity is directly associated with the production of antibiotics, such as DAPG, PHZs, PRN, PLT, and other hydrolytic enzymes. Advanced knowledge on plant protection features of *P. fluorescens* antagonists such as genes contributing to rhizosphere competence and suppressing plant diseases, along with factors affecting root colonization, is required for the management of phytopathogens.

## 15 Future Prospective

Sustainable agriculture is the basic requirement of the world in current times because of the harmful consequences of chemicals used in agriculture. However, a knowledge gap still exists regarding plant–microbial interactions under different stress circumstances, mainly biotic stress. Currently, various products in the market are advertised and sold as biofertilizers and biopesticides. Due to the need to decrease the use of chemical fertilizers, the world’s demand for more of such products and organically grown food is increasing. The positive effects have already been mentioned, and the development and production of biofertilizers and biopesticides could be an appropriate solution for developing countries, which badly suffer from shortages of food. The great potential for the use of biological products in agriculture and food production is clear because it contributes to agricultural sustainability and gives solutions to issues regarding the environmental impact of existing agricultural practices. However, future research demands rhizo-engineering based on the favorable identification and partitioning of new biomolecules, which may make the distinctive settings for interactions between plants and microbes. The application and investigation of multistrain bacterial inoculants over a single strain could be an effective means of disease suppression and management. Moreover, genetic modifications for increasing the biocontrol efficiency of microbes can also be an emerging research field for future disease management. For example, the transformation of strains with augmented levels of antimicrobial activity and growth-increasing metabolites can be good options.
